# Software for Matching Standard Activity Enzyme Biosensors for Soil Pollution Analysis

**DOI:** 10.3390/s21031017

**Published:** 2021-02-02

**Authors:** Valentina A. Kratasyuk, Elizaveta M. Kolosova, Oleg S. Sutormin, Viktoriya I. Lonshakova-Mukina, Matvey M. Baygin, Nadezhda V. Rimatskaya, Irina E. Sukovataya, Alexander A. Shpedt

**Affiliations:** 1 Department of Biophysics, Institute of Fundamental Biology and Biotechology, Siberian Federal University, 79 Svobodny pr., 660041 Krasnoyarsk, Russia; VKratasyuk@sfu-kras.ru (V.A.K.); OSutormin@sfu-kras.ru (O.S.S.); VLonshakova-Mukina@sfu-kras.ru (V.I.L.-M.); NRimatskaya@sfu-kras.ru (N.V.R.); ISukovataya@sfu-kras.ru (I.E.S.); 2 Federal Research Center ‘Krasnoyarsk Science Center SB RAS’, Photobiology Laboratory, Institute of Biophysics, Russian Academy of Sciences, Siberian Branch, 50/50 Akagemgorodok, 660036 Krasnoyarsk, Russia; 3 Department of High-Efficiency Calculations, Siberian Federal University, 26-ULK building Kirensky St., 660074 Krasnoyarsk, Russia; mbaygin-ki20@stud.sfu-kras.ru; 4 Federal Research Center ‘Krasnoyarsk Science Center SB RAS’, Krasnoyarsk Research Institute of Agriculture, Russian Academy of Sciences, Siberian Branch, 66 Svobodny pr., 660037 Krasnoyarsk, Russia; ashpedt@sfu-kras.ru; 5 Department of Aquatic and Terrestrial Ecosystems, Institute of Fundamental Biology and Biotechology, Siberian Federal University, 79 Svobodny pr., 660041 Krasnoyarsk, Russia

**Keywords:** biosensors, enzyme, butyrylcholinesterase, lactic dehydrogenase, bacterial luciferase, soil pollution, software

## Abstract

This work is dedicated to developing enzyme biosensor software to solve problems regarding soil pollution analysis. An algorithm and specialised software have been developed which stores, analyses and visualises data using JavaScript programming language. The developed software is based on matching data of 51 non-commercial standard soil samples and their inhibitory effects on three enzyme systems of varying complexity. This approach is able to identify the influence of chemical properties soil samples, without toxic agents, on enzyme biosensors. Such software may find wide use in environmental monitoring.

## 1. Introduction

Enzyme biosensors are characterised by a high level of specificity and sensitivity to the analyte, which has led to their wide use for the detection of various chemical and biological substances in clinical trials, in food and in environmental monitoring [1,2]. Currently, biosensors are mainly used in the determination of pesticides (primarily organophosphorus compounds and carbamates) and heavy metal salts [3]. We previously showed the potential use of enzyme systems (bioluminescent and non-bioluminescent) of varying complexity as indicator systems to evaluate the level of pollution of soil samples [4]. The mechanism of actuation of environmental contaminants on the activity of the enzyme systems is based on the detection of fluctuation of enzymatic activities in the presence of the contaminants with respect to the control. The fluctuation can be as specific as non-specific effects [5], but the bioluminescent enzyme systems usually show integral effects, since they are qualitative assays [6]. It has been shown that the bioluminescent enzyme system has sensitivity to heavy metal salts and nanomaterials [4,5,7]. Also butyrylcholinesterase has demonstrated the high sensitivity to carbamate pesticides [4,8] Besides, studies have revealed the difficulties of enzymatic bioassay of soils associated with the active influence of soil extracts on both the activity of enzymatic systems and their interaction with toxic agents [7]. This problem is also relevant for other bioassay methods that use biosensors [9]. It is likely that the effects caused by the complexity of the studied environment can be determined by comparing it with a reference (standard, uncontaminated) soil sample, similar to the approaches used in chemical testing. Unfortunately, to date, there is no consensus on which soils can be utilized as a standard. The first approach is the use of a model soil (MS) developed for earthworm bioassay [10]. Despite the advantages of this approach, MS does not reflect the entire variety of soils and their properties, on which the distribution of pollutants and their bioavailability depend [11,12,13]. In addition, the possible influence of the parameters of the soil itself (without toxic agents) on the test objects is not considered [14].

The literature describes several approaches for the formalisation of these results of soil condition assessment. One such approach is the toxicity test battery integrated index, which is defined as the average of all bioassay responses with different weight coefficients [15]. For determining toxicological parameters according to the degree of inhibition by toxic agents, Ecoscores were suggested [16]. The so-called triad method takes into account the data from chemical testing, bioindication and toxicological testing to determine the integral index of the soil status. Chemical substances, bioassay function value and bioindication function value in the sample are used as indicators [17]. A significant limitation of the above approaches is that it is necessary to know the concentration of toxic agents in soils to calculate soil indices, which is not always possible to determine, especially if it is not specific, but an integral bioassay method is used. The most promising method for storing, identifying and managing soil sample data using integral bioassay methods is a system based on barcode and WebGIS technology. Such systems are based on the principle of a global database, with each sample being assigned an individual number (barcode) [18] and are convenient tools.

To sum up, there is still a lack of interpretation tools results received by studying highly complex samples. A logical development of a bioassay methodology system using biosensors is to combine the above approaches—standard soils for comparison and software for their analysis and visualisation—into a single tool. Therefore, this study aimed to develop a novel approach for improving and interpreting the results of enzymatic bioassays that is applicable to the analysis of complex environments using standard soil samples. In this study, we describe the development of a specialised enzymatic biosensor software.

## 2. Materials and Methods

### 2.1. Basis for Software Development

The software product was based on the Euclidean distance (ED) method Equation (1). A prerequisite for the new approach to work is the availability of standard soil samples, which are matched to each new test sample. The search algorithm finds the minimum difference between the test sample and the standard sample using Equation (1).

(1) $$d\left(p,q\right)=\sqrt{{\left(p_{1}-q_{1}\right)}^{2}+{\left(p_{2}-q_{2}\right)}^{2}+\cdots +{\left(p_{n}-q_{n}\right)}^{2}}=\sqrt{\overset{n}{\underset{k=1}{\sum }}{\left(p_{k}-q_{k}\right)}^{2}}$$

The requirements for the software are as follows: (1) it should contain information regarding the soil characteristics presented in Table 1 and (2) it should allow working with data in any Internet browser independently from the operating system and platform used.

### 2.2. Experiment Scheme and Determination of Soil Characteristics of Standard Soil Substrates

**Figure 1:**
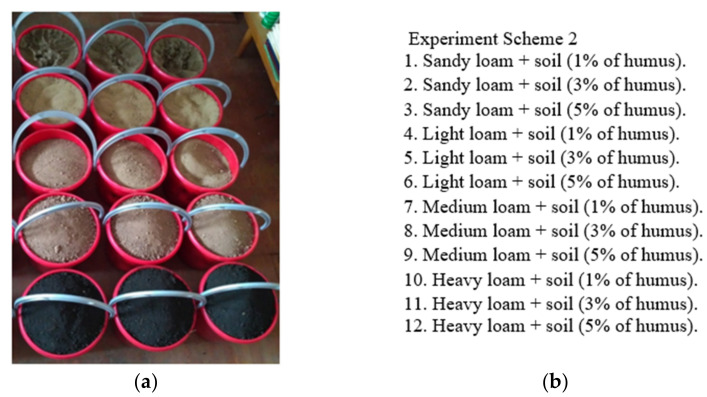
Standard soils: (**a**) view of vessels containing basic soil substrates, (**b**) the experiment scheme based on the basic soil substrates.

To develop software for matching standard activity enzyme biosensors for soil pollution analysis was prepared 51 soil samples, which was collected from the special forest-steppe zones of Krasnoyarsk region, Russia. Complete factorial experiment schemes included variants of types and varieties of soils and minerals with different particle size distribution and humus content. Based on the five basic soil substrates Figure 1a (sandy loam, light, medium and heavy loam, high-humus soil), 12 varieties of soil substrates were designed, each differing in particle size distribution and humus content Figure 1b.

The organic matter (humus), pH of the soil solution, and the particle size distribution of the soil were determined according to previously described methods [19,20,21]. The soil organic matter (humus) is determined by modified Tyurin photometric method. Soil samples are sieving through a 2.5 mm sieve and removal of visible roots and plant residues. The soil organic matter is oxidized by potassium dichromate solution with sulphuric acid and heated at boiling point for 1 h. After cooling the suspension is washed with water, agitated and left to settle. The soil organic matter is established photometrically (λ 590 nm). The mass of organic matter in the analyzed sample is determined by the calibration graph. The pH of the soil solution is determined by a glass electrode in a 1:2.5 (volume fraction) suspension of soil in 1 mol/L potassium chloride solution (pH in KCl). The particle size distribution of the soil is determined by standard pipette analysis (similarly to ISO 11277:2002-08, 2002).

The optical density of water soil extracts (1:5 volume fraction) to determine the total amount of solute was determined at a wavelength of 250 nm (D250) using a Cary 5000i spectrophotometer (Agilent Technologies).

### 2.3. Enzymatic Assay

The butyrylcholinesterase (BChE) activity was determined using the Ellman method as follows: the following components were sequentially introduced into the spectrophotometer cuvette: 800 μL of 0.05 M potassium phosphate buffer (pH 7.9); 200 μL of sample (control or test solution); 30 μL of BChE (0.11 U); 57 μL of 0.2 mM 5-5’dithiobis-2 (nitrobenzoic acid) solution and 57 μL of 2 mM S-BCh-I solution.

The optical density of the solution was recorded over 5 min at 412 nm using a UV-2600 spectrophotometer (Shimadzu, Japan). The change in optical density was used to calculate the rate of hydrolysis of the BChE substrate, and from the obtained results, the relative BChE activity (A) in the reaction mixture was determined.

To assess the effect of the inhibitor on BChE activity, the residual BChE activity (OA) in the reaction mixture was calculated using the following formula OA = (A/A_0_) * 100%, where A is the rate of S-BCh-I BChE hydrolysis in the test solution and A_0_ is the rate of S-BCh-I BChE hydrolysis in control solution.

To determine the activity of the conjugated system NADH:FMN-oxidoreductase + luciferase (R + L), a reaction mixture of the following composition was used: 300 μL of 0.05 M potassium phosphate buffer (pH, 6.8); 5 μL of analytical bioluminescence reagent kit solution; 50 μL of 0.0025% tetradecanal solution; 100 μL of 0.4 mM NADH solution; 50 μL of distilled water (control) or test solution and 10 μL of 0.5 mM FMN solution. One flask of the analytical bioluminescence reagent kit solution (Laboratory of Nanobiotechnology and Bioluminescence, Institute of Biophysics, Siberian Branch, Russian Academy of Sciences, Krasnoyarsk) contained 0.5 mg of luciferase obtained from recombinant E. coli strain and 0.18 units of activity of NADH:FMN oxidoreductase from Vibrio fischeri. Enzyme solutions were prepared in 0.05 M potassium phosphate buffer (pH, 6.8).

The kinetic parameters of the bioluminescent trienzyme system lactate dehydrogenase + NADH:FMN-oxidoreductase + luciferase (LDH + R + L) were measured in a mixture containing the following components: 300 μL of 0.05 M potassium phosphate buffer (pH, 6.8); 5 μL of 0.5 mg/mL lactate dehydrogenase solution; 10 μL of analytical bioluminescence reagent kit solution; 10 μL of 15 mM lactate solution; 100 μL of 0.5 mM NAD+ solution; 50 μL of 0.0025 % tetradecanal solution; 10 μL of 0.5 mM FMN solution in distilled water and 50 μL of distilled water (control) or test solution. The reaction was initiated with the addition of FMN solution.

For two- and three-enzyme systems, the reaction mixture was placed in a luminometer cuvette (PromegaGloMax 20/20 Luminometer, USA), and the luminescence intensity was measured over 300 s. The residual luminescence was calculated according to the formula (I/I_0_) × 100%. At I/I_0_ > 80%, the analysed soil sample was considered to have no impact, at 50% < I/I_0_ < 80%, the analysed soil sample was considered as having an impact, and at I/I_0_ < 50%, the analysed soil sample was considered to have a significant impact.

## 3. Results and Discussion

Difficulties in interpreting the results of studying the composition of complex environments can be resolved by using reference standard samples. With this approach, the intrinsic influence of the sample is taken into account when examining samples. The success of the search for a standard directly depends on the measurement and approximation method. In most cases, ED Equation (1) is used to find the distance between objects with quantitative characteristics and the same dimensions. The input data is an array of vectors m × n, where m is the number of characteristics of a standard soil sample and n is the number of standards. For the convenience of presentation, the data were reconstructed to the form of a matrix Equation (2):

(2) $$X=\left[\begin{array}{cccc} x_{11} & x_{12} & \cdots & x_{1n}\\ x_{21} & x_{22} & \cdots & x_{2n}\\ \vdots & \vdots & \ddots & \vdots \\ x_{m1} & x_{m2} & \cdots & x_{mn} \end{array}\right]$$

Another input is the data for one test soil sample, which are converted into one column vector of dimensionality m × 1

(3) $$Y=\left(\begin{array}{c} y_{1}\\ y_{2}\\ \vdots \\ y_{n} \end{array}\right)$$

**Figure 2:**
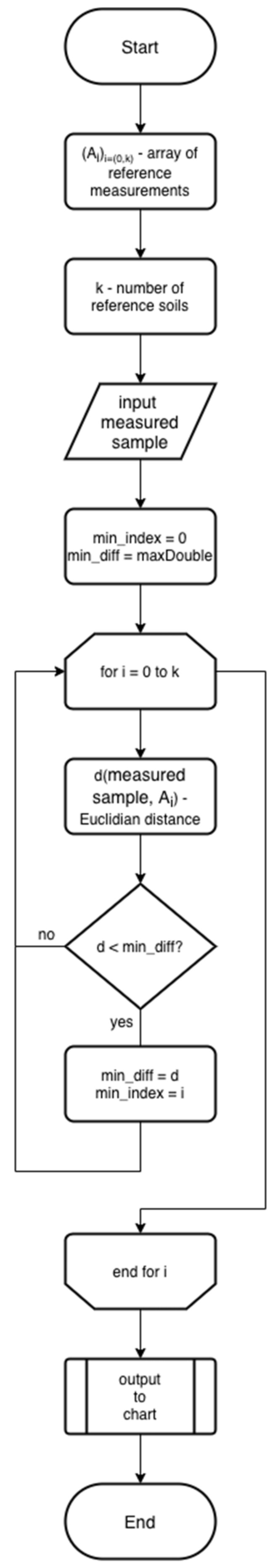
Flowchart of the search of the reference with a minimum deviation using Euclidean Distance.

The process of finding the closest possible standard sample is as follows Figure 2:

1. The program reads reference soil’s data from a *.js file with json object inside.
2. The number of reference soils (k) is estimated.
3. The program reads sample soil’s data from the program’s input box.
4. Sample‘s index is equating to zero. The minimum deviation is equal to the maximum possible number.
5. Loop through each element of the source data array.
6. Based on Equation (1), the ED is calculated for the reference and investigated soils.
7. If calculated ED is lower than minimum deviation, minimum deviation is equating to ED. Index of minimum reference deviation is equating to current loop value.
8. According to the obtained index from the array of reference soil, all the data for the most suitable reference soil are obtained.

The output of the algorithm is the tested standard sample. Before graphically displaying the data, the data is made uniform using the formula y = y_i_/y_max_ × 100%. Bringing all data on one scale allows us to more accurately assess the contribution of each parameter, displaying them in one graph in one scale. To fill the database of the application with information regarding standard soils and their effect on enzymatic systems, 51 samples of conditionally pure model soils were examined. Soil samples have different particle size distributions, exchange acidity and soil organic matter contents, which varies widely, from 0.37% to 8.50%, with increments of 1%, 3% and 5% (see Table S1). The content of physical clay ranged from 8.6% to 47.5%, and the varieties were represented by cohesive sand, sandy loam and light, medium and heavy loams. The pH (in KCl) of salt suspension also differed, from 5.45 to 8.00 units, which indicates a weakly acidic, close to neutral, neutral, slightly alkaline and alkaline reaction of the environment. Analysis of all the obtained data allows to assert that the tested used a wide variety of soils and rocks with a wide range of variation of basic properties.

The effect of water extracts from standard soil-based soils on single-, two- and three-enzyme systems was studied. For the BChE system, no significant inhibition by soil extracts was observed, as the % OA parameter remained at least 95%. For some samples, the opposite activation effect was observed (an increase in OA relative to the control), in some cases OA level reached 155%. The residual fluorescence T2, % of the two-enzyme system was in the range of 49% to 103%. The greatest inhibiting effect was shown by soil samples from groups with a high humus content and a heavier particle size distribution. The residual luminescence of the three-enzyme system T3, % varied in the range of 22% to 100%, which indicates its high sensitivity to soil components. The D250 optical density value ranges from 0.16 to 1.59. There is a clear pattern sample group effect: the heavier the soil in terms of particle size distribution, the higher the optical density of the extract from this soil. The above measured parameters in combination with soil characteristics humus content, pH and particle size distribution, see Table 1, have been entered into the application. These standards have been collected, described, analysed, and then categorised, generalised and added to the general database. Thus, the database consists of 51 reference soil samples, divided into 17 type categories (legend for soil categories). Each category has three different reference examples. An example of input data file structure is shown in Table 2.

An application has been developed in the JavaScript programming language based on the algorithm Figure 2. When the user enters experimental data, the application finds a data record on the reference sample and passes the data about the standard and the test sample to the chart library (chart.js). Afterwards, the found standard sample and the test sample are displayed on a single graph.

**Figure 3:**
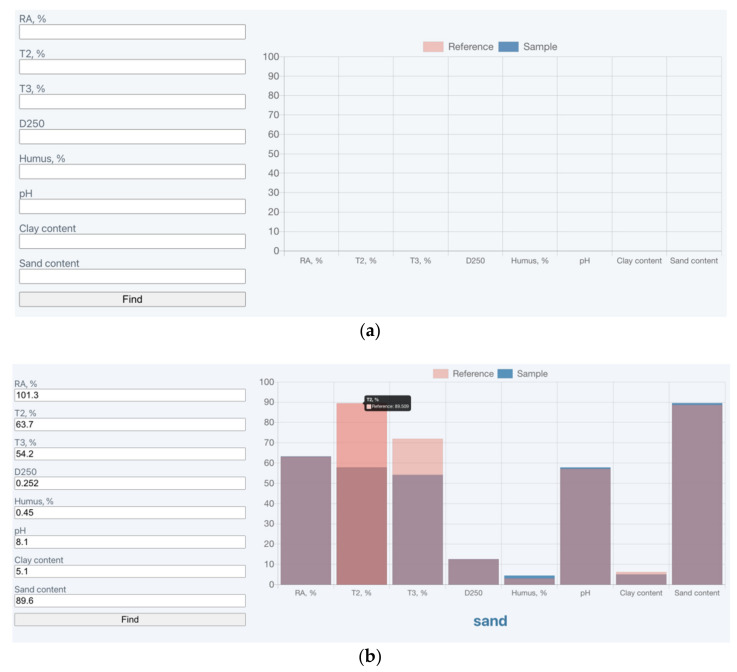
View of the application: (**a**) when launched in a browser; (**b**) when completing the input boxes and selecting a suitable standard soil.

Visually, the application includes input boxes, an Output Data button and a chart area. When the application starts, the input boxes are not filled in Figure 3a. The user of the application (the researcher) enters the experimentally obtained characteristic measurement data of the test soil into the corresponding boxes. After the user clicks the “Find” button, the software searches for the appropriate reference standard based on the previously described algorithm. After the search, the application outputs a chart and, in the lower part of the chart, the code name of the standard soil. The chart simultaneously displays soil characteristic values for the standard sample (pink bars) and the test soil (blue bars). Of note, the data on the characteristics of the test soil and the standard soil are unified, that is, they are brought to one scale, where the maximum value of the standard sample characteristic is taken as 100%. This unification is due to the fact that the percentage deviation of the test sample from standard is more informative than a specific absolute value. For convenience of comparison, value overlap is shown in violet. In addition, on mouse cursor hover over any of the bars, its numerical value is shown Figure 3b.

**Figure 4:**
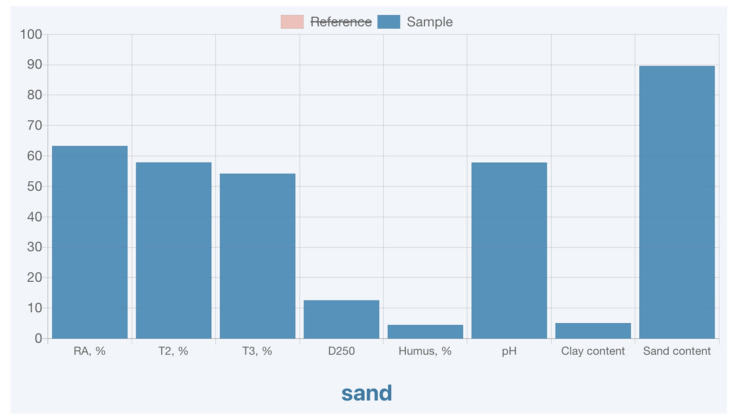
Application graph, the “reference” category is not active (crossed out) and the data is only displayed for “sample”.

The application can also display the bars of the standard and test sample separately, which can be switched on and off by selecting the appropriate category in the legend at the top of the chart Figure 4.

A clear advantage of our application is that it already contains the data on various standard soils and allows us to match a reference standard based on soil characteristics of test samples. The design of the application allows us to collect and analyse soils characteristic of different territories and enter them into the application’s database as standard soil samples.

## 4. Conclusions

We have developed specialised enzyme biosensor software for soil pollution analysis. This application contains information on the characteristics of standard soils and the effect of aqueous extracts from these soils on three enzymatic systems. This information can be considered as having a background inhibitory effect on the components of the biological module of the biosensor produced by soil samples, which is a valuable component. This software may find wide use in environmental monitoring.

## Figures and Tables

**Table 1:** Summary of characteristics of the soil samples.

#	Characteristic Abbreviation	Description	Unit
1	RA	Residual activity of BChE	A/A_0_, %
2	T2	Residual luminescence value of Red + Luc system	I/I_0_, %
3	T3	Residual luminescence value of LDH + Red + Luc system	I/I_0_, %
4	D250	Optical density of an aqueous extract from soils at a wavelength of 250 nm	Optical density units
5	Humus	Mass fraction of organic matter (humus)	%
6	pH	pH (in KCl) of soil solution	pH units
7	Clay content	Physical clay fraction (0.001 mm, silt)	%
8	Sand content	Physical sand fraction percentage (0.05–0.25 mm fine sand)	%
9	Sample name	Name of the standard sample from the database with measured values for comparing the data of the studied soil	

**Table 2:** An example of input data.

RA	T2	T3	D250	Humus	pH	Clay	Sand	Sample Name
108.79	90.78	78.55	0.25	0.38	8	6.7	90.4	sand
101.09	102.81	86.81	0.16	0.33	7.9	5.9	85.4	sand
101.09	98.46	71.99	0.25	0.31	8	6.3	88.7	sand
104.95	101.59	71.93	0.48	0.55	5.3	8.5	81.7	light loam

Note: RA-Residual activity of BuChE (A/A_0_, %), T2-The residual luminescence value of Red + Luc system (I/I_0_, %), T3-The residual luminescence value of LDH + Red + Luc system (I/I_0_, %), D250-Optical density of an aqueous extract from soils at a wavelength of 250 nm (Optical density units), Humus-Mass fraction of organic matter (humus) (%), pH-pH of soil solution (pH units), Clay content-Physical clay fraction percentage (0.001 mm, silt) (%), Sand content-Physical sand fraction percentage (0.05–0.25 mm fine sand) (%), Sample name-Name of the standard sample from the database with measured values for comparing the data of the studied soil.

## Data Availability

The program could be downloaded on this website: https://github.com/actimel35/barcode.

## Supplementary Materials

The following are available online at https://www.mdpi.com/1424-8220/21/3/1017/s1, Table S1: Properties of tested standard soils, which is essential for soil studies.

## References

[1] Amine A.H., Mohammadi H., Bourais I., Palleschi G. (2006). Enzyme inhibition-based biosensors for food safety and environmental monitoring. Biosens. Bioelectron..

[2] Pundir C.S., Malik A. (2019). Bio-sensing of organophosphorus pesticides: A review. Biosens. Bioelectron..

[3] Bachan Upadhyay L.S., Verma N. (2013). Enzyme inhibition based biosensors: A review. Anal. Lett..

[4] Kolosova E.M., Sutormin O.S., Esimbekova E.N., Lonshakova-Mukina V.I., Kratasyuk V.A. (2019). Set of enzymatic bioassays for assessment of soil contamination. Dokl. Biol. Sci..

[5] Esimbekova E.N., Nemtseva E.V., Bezrukikh A.E., Jukova G.V., Lisitsa A.E., Lonshakova-Mukina V.I., Rimatskaya N.V., Sutormin O.S., Kratasyuk V.A. (2017). Bioluminescent enzyme inhibition-based assay to predict the potential toxicity of carbon nanomaterials. Toxicol. In Vitro.

[6] Esimbekova E., Kratasyuk V., Shimomura O., Thouand G., Marks R. (2014). Application of enzyme bioluminescence in ecology. Bioluminescence: Fundamentals and Applications in Biotechnology.

[7] Sutormin O.S., Kolosova E.M., Nemtseva E.V., Iskorneva O.V., Lisitsa A.E., Matvienko V.S., Esimbekova E.N., Kratasyuk V.A. (2018). Enzymatic bioassay of soil: Sensitivity comparison of mono-, double- and triple-enzyme systems to soil toxicants. Tsitologiya.

[8] Arduini F., Ricci F., Tuta C.S., Moscone D., Amine A., Palleschi G. (2006). Detection of carbamic and organophosphorous pesticides in water samples using a cholinesterase biosensor based on Prussian Blue-modified screen printed electrode. Anal. Chim. Acta.

[9] Semenov A.M., Sokolov M.S. (2010). The concept of soil health: Fundamental and applied aspects of the justification of the evaluation criteria. Agrokhimiya.

[10] OECD Guidelines for the Testing of Chemicals, Section 2.

[11] Van Gestel C.A.M. (2012). Soil ecotoxicology: State of the art and future directions. ZooKeys.

[12] Davies N.A., Hodson M.E., Black S. (2003). Is the OECD acute worm toxicity test environmentally relevant? The effect of mineral form on calculated lead toxicity. Environ. Pollut..

[13] Criel P., Lock K., Van Eeckhout H., Oorts K., Smolders E., Janssen C.R. (2008). Influence of soil properties on copper toxicity for two soil invertebrates. Environ. Toxicol. Chem..

[14] Amorim M.J., Novais S., Römbke J., Soares A.M. (2008). Avoidance test with *Enchytraeus albidus* (Enchytraeidae): Effects of different exposure time and soil properties. Environ. Pollut..

[15] Prato E., Parlapiano I., Biandolino F. (2015). Ecotoxicological evaluation of sediments by battery bioassays: Application and comparison of two integrated classification systems. Chem. Ecol..

[16] Lors C., Ponge J.F., Aldaya M.M., Damidot D. (2011). Comparison of solid and liquid-phase bioassays using ecoscores to assess contaminated soils. Environ. Pollut..

[17] Terekhova V.A., Pukalchik M.A., Yakovlev A.S. (2014). The triad approach to ecological assessment of urban soils. Eurasian Soil Sci..

[18] Guo W., Yi X., Chen Y., Wang X. (2010). Soil spatial information management system based on WebGIS and barcode technology. Trans. Chin. Soc. Agric. Eng..

[19] GOST 12536-2014, Soils. Methods of Laboratory Granulometric (Grain-Size) and Microaggregate Distribution. https://www.russiangost.com/p-138335-gost-12536-2014.aspx.

[20] GOST 26483-85. Soils. Preparations of Salt Extract and Determination of Its pH by CINAO Method. https://www.russiangost.com/p-50090-gost-26483-85.aspx.

[21] GOST 26213-91. Soils. Methods for Determination of Organic Matter. https://www.russiangost.com/p-52750-gost-26213-91.aspx.

