# Supplementary material for: Software for Matching Standard Activity Enzyme Biosensors for Soil Pollution Analysis
Source: Sensors (Basel). 2021 Feb 2;21(3):1017. doi: 10.3390/s21031017 (PMC7867351; doi:10.3390/s21031017)
Supplement: Supplementary file 1 [file sensors-21-01017-s001.pdf]

Supplementary Materials

Table S1: Properties of tested standard soils, which is essential for soil studies (three samples in each group).

Abbreviation of soil sample	pH (in KCl)	Soil organic matter, %	Physical sand fraction percentage (0.05–0.25 mm fine sand)	Physical clay fraction (0.001 mm, silt)
	8	0.38	90.4	6.7
Sandy loam	7.9	0.33	85.4	5.9
	8	0.31	88.7	6.3
	5.3	0.55	81.7	8.5
Light loam	5.7	0.24	79.1	8.9
	5.2	0.38	77.4	8.3
	7.3	0.48	57.3	17.3
Medium loam	7.6	0.38	59.4	15.7
	7.3	0.56	58.9	16.8
	7.5	0.73	28.3	26.4
Heavy loam	7	0.81	29.1	29.2
	7.2	1.01	24.5	29.2
	6.3	8.37	17.6	21
high humus soil	7	7.96	19.1	20.4
	6.8	8.87	18.7	20.9
	7.8	0.55	84.8	6.2
Sandy loam, 1%	6.9	1.34	84	9.8
	6.9	1.04	84.5	7
	6.7	4.72	67.9	12.8
Sandy loam, 3%	6.7	2.26	69.2	13.1
	6.8	1.95	79.4	9.7
	6.7	5.32	53.4	16.9
Sandy loam, 5%	6.8	3.09	74.3	10.8
	6.7	4.21	68	13
	6.6	1.24	77.3	8.4
Light loam, 1%	6.4	1.58	77.1	9
	6.3	1.33	75.6	9.8
	7	2.5	70.3	9.7
Light loam, 3%	6.3	3.46	59	14.2
	6.2	1.84	70	11.5
	6.3	5.22	54.1	16.1
Light loam, 5%	6.3	3.5	58	12.9
	6.8	4.01	58.1	14.1
	6.3	1.52	60.6	13.9

**Abbreviation of soil sample**	**pH (in KCl)**	**Soil organic matter, %**	**Physical sand fraction percentage (0.05–0.25 mm fine sand)**	**Physical clay fraction (0.001 mm, silt)**
Medium loam, 1%	7.1	1.2	58.4	16.1
	6.5	0.86	59	15.1
	6.8	1.81	54.1	16.6
Medium loam, 3%	6.8	3.32	53	16.2
	6.8	2.81	53.6	16.2
	7	3.5	48.3	18.2
Medium loam, 5%	6.9	4.92	45.8	17.2
	6.9	5.22	43.8	18.7
	7.1	2.69	26.6	27.8
Heavy loam, 1%	7.2	1.84	29.2	27.1
	7.2	1.64	25.7	28.7
	7.2	4.01	25	28.2
Heavy loam, 3%	7.2	2.76	24	26.1
	7	4.03	24.4	23.8
	7.2	5.42	21.6	25
Heavy loam, 5%	7.1	4.92	23.1	24.2
	7.1	6.03	21.5	26.6
